# Assessment of nutritional status of oncology patients at hospital admission: A Portuguese real-world study

**DOI:** 10.3389/fnut.2022.972525

**Published:** 2022-09-09

**Authors:** Carolina Trabulo, Joana Lopes, David da Silva Dias, João Gramaça, Isabel Fernandes, Rita Gameiro, Idília Pina, Antti Mäkitie, Faith Ottery, Paula Ravasco

**Affiliations:** ^1^Centro Hospitalar do Barreiro Montijo, Barreiro, Portugal; ^2^Centre for Interdisciplinary Research in Health (CIIS), Universidade Católica Portuguesa, Lisbon, Portugal; ^3^Hospital Universitário Algarve, Faro, Portugal; ^4^Research Program in Systems Oncology, Faculty of Medicine, University of Helsinki, Helsinki, Finland; ^5^Division of Ear, Nose and Throat Diseases, Department of Clinical Sciences, Intervention and Technology, Karolinska Institute and Karolinska University Hospital, Stockholm, Sweden; ^6^Department of Otorhinolaryngology-Head and Neck Surgery, Helsinki University Hospital and University of Helsinki, Helsinki, Finland; ^7^Católica Medical School, Universidade Católica Portuguesa, Lisbon, Portugal; ^8^Clinical Research Unit, Centro de Investigação Interdisciplinar Egas Moniz (CiiEM), Egas Moniz, Cooperativa de Ensino Superior, CRL, Almada, Portugal

**Keywords:** scored patient-generated subjective global assessment, nutritional assessment, malnutrition, oncology, subjective global assessment (SGA), cancer, patient admission

## Abstract

**Background:**

Nutritional status in patients with cancer has a determining role in the evolution of the disease and tolerance to treatments. Severity of undernutrition impacts morbidity and mortality in cancer patients and can limit patient response to the optimal therapies if nutritional issues are not appropriately addressed and managed. Despite the importance of malnutrition for the clinical evolution of oncology patients, there is not yet a universally accepted standard method for evaluating malnutrition in such patients. The aim of this study was to stratify the nutritional status of inpatients at an Oncology Department.

**Methods:**

This is an observational study with 561 cancer patients, assessed at admission to a Medical Oncology Department from November 2016 to February 2020. All patients were considered eligible. Non-compliant and/or comatose patients were excluded. Nutritional status was assessed using the PG-SGA, BMI classified with the WHO criteria, and calculation of the percentage of weight loss in the previous 3–6 months.

**Results:**

A total of 561 patients (303 F: 258 M; mean age 65 ± 13 years) were included. One-third of the patients, n=191/561 (34%), lost 6% of their weight in the month prior to admission and 297/561 (53%) patients lost 10.2% of weight in the previous 6 months. Mean BMI was 24.1 ± 5.8 kg/m^2^; *N* = 280/561 (50%) patients had regular BMI according to the WHO criteria. *N* = 331/561 (59%) patients reported eating less in the month prior to admission. *N* = 303/561 (54%) had moderate/severe deficits of muscle and adipose compartments. The PG-SGA identified 499/561 (89%) patients as moderately/severely malnourished, of which 466/561 (83%) patients scored ≥9 points, meeting criteria for a critical need for nutritional support. Fifteen percent of patients scored >4 points, indicating a need for directed therapy for symptom control and only 1% scored <2 points (maintenance nutritional counseling).

**Conclusion:**

In this oncological setting, a higher proportion of patients were nutritionally-at-risk or with moderate/severe malnutrition. The large majority of patients in this study presented with a critical need for nutritional intervention. These findings highlight the need for an integrated assessment of nutritional status at patient referral. This will allow early and timely nutrition care, which is recommended to prevent or reverse further deterioration of the condition and to optimize treatment administration.

## Introduction

The incidence of malnutrition amongst patients with cancer ranges between 40 and 80% (1). These patients are particularly susceptible to nutritional depletion due to the physical and metabolic effects of cancer, as well as anticancer therapies. Severity of undernutrition is a major source of morbidity and mortality in cancer patients and its presence can limit patient response to even the best therapies if nutritional issues are not appropriately addressed and managed (1–3).

Unintentional weight loss is experienced in the majority of patients with gastroesophageal, pancreatic, head and neck and lung cancer. There is also a high prevalence of weight loss in patients with advanced disease such as advanced colorectal cancer (4–6).

Compromised nutritional status can adversely impact both the quantity and quality of survival and survivorship. Reports have shown that weight loss is an important predictor of decreased survival (7, 8). Chemotherapy patients have a reduced quality of life (9, 10), a higher frequency of hospital readmission, and a longer hospital stay if they are malnourished at baseline or during oncological therapy (11). It is estimated that 4–23% of cancer patients with incurable disease may eventually die because of progressive malnutrition (12). This knowledge highlights the association of malnutrition and body compositional deficit with dose-limiting toxicity (DLT), which prevents the ability to achieve optimal treatment on time and at a full dose.

Malnutrition among patients with cancer is driven by inadequate food intake, decreased physical activity and catabolic derangement in metabolism (13). Nutritional treatment of undernourished patients has been linked with better outcomes (13, 14). Evidence shows that it is paramount to have early and proactive identification of cancer patients at high nutritional risk, to allow for comprehensive nutritional assessment, establish the level of deficit and implement a clinically appropriate intervention (15). This comprehensive approach to nutrition care may lead to improvements in nutritional status, quantity and quality of life, patient satisfaction and treatment outcomes (16).

The use of standardized and validated tools, is recommended globally (17–19) for all patients admitted to hospital, and often times required for hospital accredidatation (20). However, in many countries, this practice is not routinely performed (19). Low awareness of malnutrition and its importance for outcomes and quality of care is a current area of concern in the oncology and nutrition communities (21–23).

An early and integrated nutritional assessment of all patients is mandatory. This may be achieved using the Patient-Generated Subjective Global Assessment (PG-SGA), which is identified in clinical practice and academic research as a reference method for the nutritional assessment of patients with chronic diseases, including cancer (24). It is recognized by the Academy of Nutrition and Dietetics as the reference method in cancer patients, allowing the identification of malnourished patients and the indication of the most appropriate type of nutritional intervention in hospital or outpatient settings. PG-SGA adequately addresses all dimensions of malnutrition as defined by the European Society for Clinical Nutrition and Metabolism (ESPEN) and the American Society for Parenteral and Enteral Nutrition (ASPEN), e.g., weight loss, food intake, symptoms, and physical function (25–28).

Our primary aim was therefore to characterize the nutritional status of patients admitted as in patients at an Oncology Department.

## Materials and methods

### Study design and setting

A prospective observational cohort study of 561 cancer patients admitted at the Medical Oncology Department of the Centro Hospitalar Barreiro-Montijo (Portugal) between November 2016 and February 2020. All patients underwent nutritional assessment by an experienced registered nutritionist in the first 48 h of admission, using the PG-SGA assessment tool. Data were obtained after informed medical consent.

The Study design and procedures were conducted in accordance with good clinical practices and were approved by the Institution Ethics, which is abided by Portuguese legislation and the Declaration of Helsinki from the World Medical Association.

### Patients

This study includes patients admitted as inpatients at the Medical Oncology department that undergo nutritional assessment by an experienced registered nutritionist, using PG-SGA assessment tool. Patients under 18 years of age, non-compliant and/or comatose, pregnant, and receiving medication that could alter basal metabolic rate were excluded. Forty patients were excluded due to significant missing data. The present study included a total sample size of 561 patients with cancer.

### Nutritional assessment

Nutritional status was assessed using the PG-SGA. The PG-SGA^®^ is a subjective nutritional assessment tool validated for use in cancer patients and hospital environments. This tool includes patient-reported information on clinical history, food intake and physical examination incorporating involuntary weight loss, changes in food intake, symptoms that could affect nutritional status and functional capacity changes. A health professional completes the questionnaire regarding the diagnosis and the relationship with nutritional needs, as well as the physical examination. For each component of the scored PG-SGA, points (0–4) are awarded depending on the impact of the symptom on nutritional status. Each item is given a score and the sum results in a nutritional status score are classified as: A—well-nourished, B—moderate malnutrition and C—severe malnutrition.

After this assessment, patients with special nutritional needs are identified and classified according to the attention needed: from 0 to 1 point, there is no need for nutritional intervention; from 2 to 3 points, the patient and his/her family require nutritional education; between 4 and 8 points, the patient requires nutritional intervention; and ≥9 points, the patient requires critical intervention and symptom control (9). The higher the score the greater the risk is for malnutrition.

Nutrition triage recommendations include patient and family education, symptom management and nutrition intervention such as additional food, oral nutrition supplements, enteral or parenteral nutrition.

This study used the translated and validated Portuguese version of the PG-SGA and its use was allowed by the PG-SGA/Pt-Global Platform (www.pt-global.org). All boxes were filled by the researchers, due to the characteristics of the study population.

Body mass index (BMI) was also calculated through the quotient between weight and height squared. Based on criteria outlined by the World Health Organization (WHO), BMI was classified into the following groups: underweight (<18.5 kg m^2^), normal (18.5–24.9 kg m^2^), overweight (25.0–29.9 kg m^2^) and obese (‡30.0 kg m^2^).

### Statistical analysis

Continuous variables were described using measures of central tendency and dispersion such as mean, and standard deviation. Categorical variables were reported as frequencies. Missing data was addressed using listwise deletion method. All data were analyzed using the IBM Statistical Package for the Social Sciences (SPSS) version 25.

## Results

**Figure 1:**
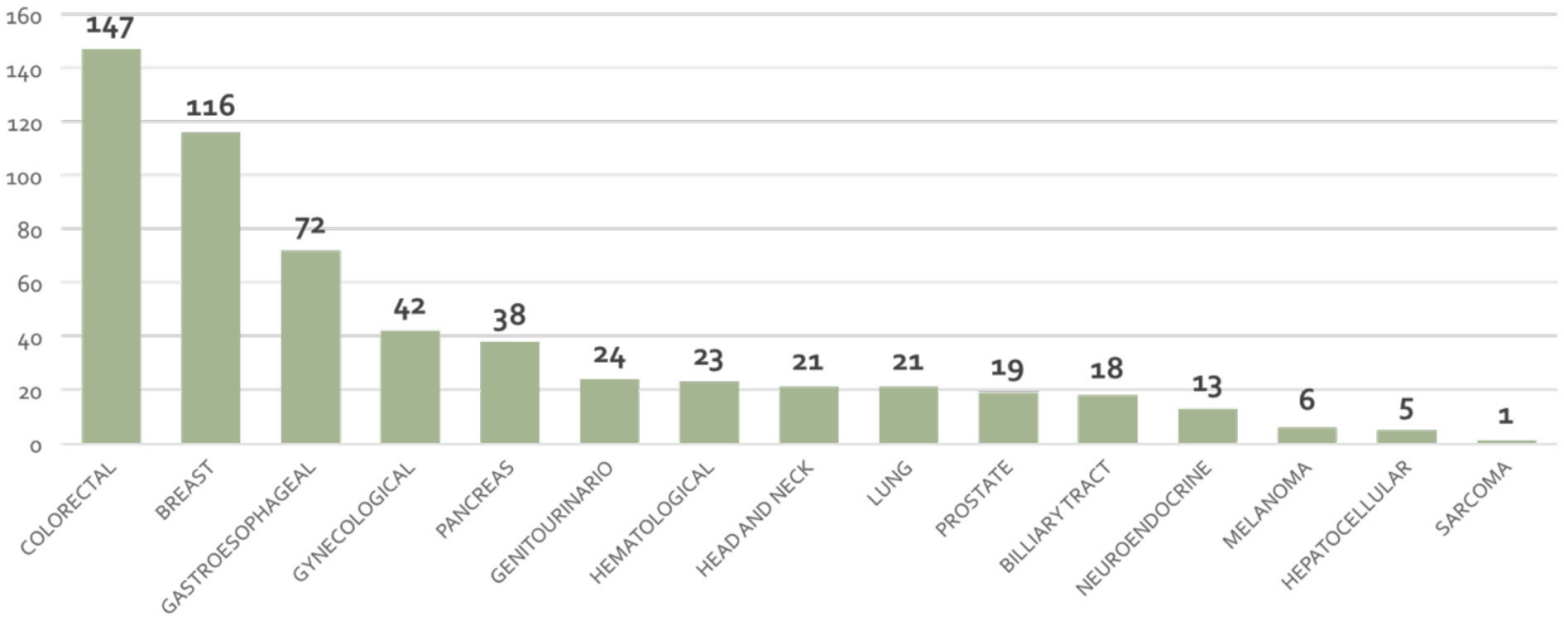
Distribution of cancer diagnoses of the series of 561 patients.

A total of 561 patients (303 women; 258 men) with a mean age of 65 ± 13 years (range, 26–91) were admitted during the four consecutive years and underwent nutritional status evaluation. Diagnosis in this study are depicted in Figure 1. The predominant diagnoses were: colorectal cancer (*n* = 147/561, 26%), breast cancer (*n* = 116/561, 18%), and gastroesophageal (*n* = 72/561, 10%) (Figure 1).

One-third of the patients (191/561, 34%), had lost weight during the month prior to the assessment, with an average weight variation of 6% (range, 0–85). We also found that 297/561 (53%) of the patients had lost weight during the previous 6 months, with an average weight loss of 10.2% (range, 0–40). In a subgroup analysis, analyzing the three most frequent types of cancers, the mean weight change over the last 6 months was more prevalent in gastrointestinal tract tumors (gastro-esophageal with a weight loss observed on average of 4% and colorectal 2%) and, on the other hand, breast cancer kept a constant weight, with a mean weight change over the last 6 months of +0.12%.

**Figure 2:**
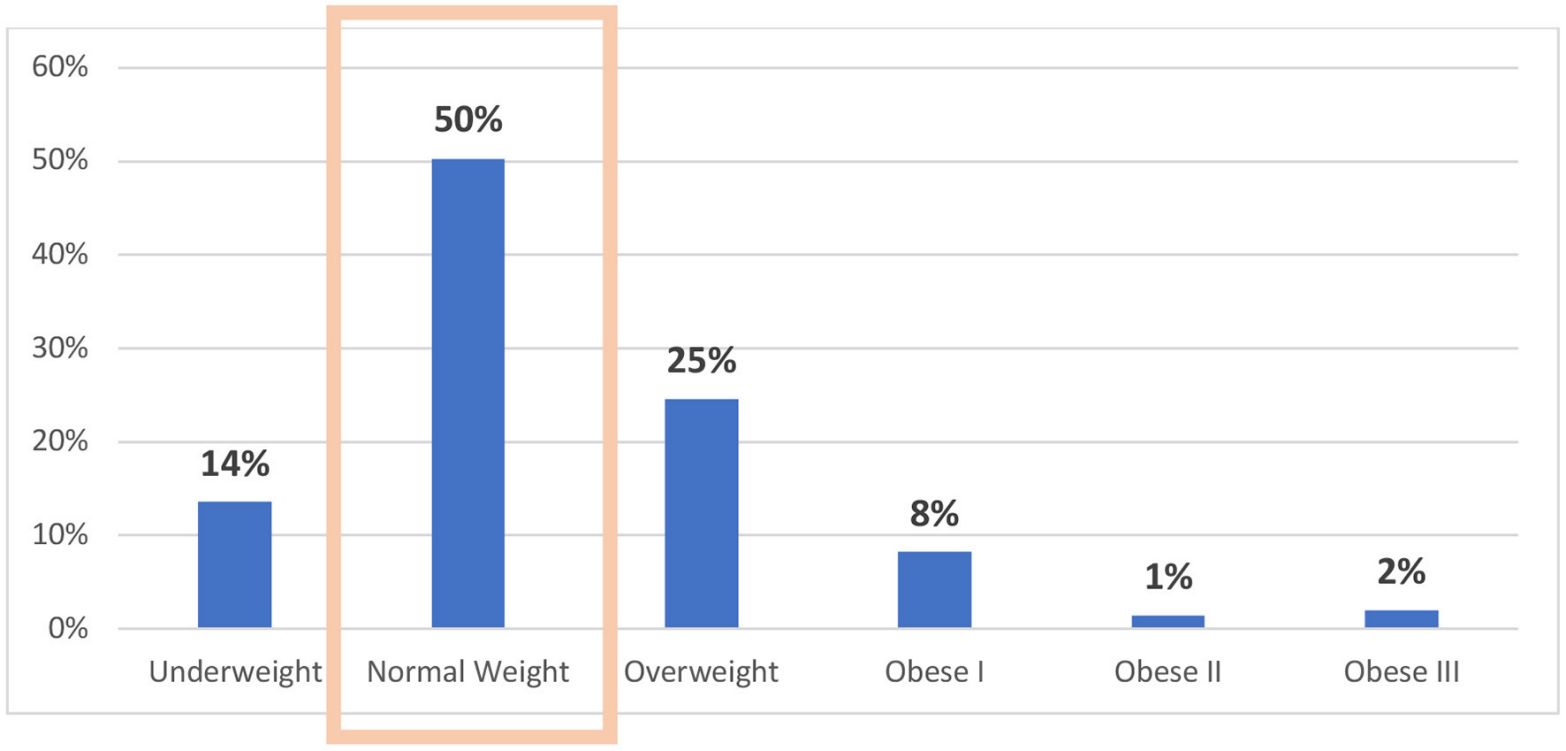
Distribution of Body Mass Index categories.

Mean BMI was 24.1 ± 5.8 kg/m^2^ and 280/561 (50%) of the patients had a normal weight according to the WHO criteria (Figure 2).

Regarding the physical examination of body components, we found that 54% of the patients presented moderate to severe deficits (grades 2 and 3) at their physical examination. More precisely, 9% presented with no deficit; 34% slight deficit; 39% moderate, and 15% presented a severe deficit.

Considering food intake during the last month, 331/561 (59%) of the patients reported eating less food as compared to their usual intake. The classification ranges from 0 to 5 where: “*I would classify my food as*, **0**—normal **1**—*normal food but less quantity* **2**—*few solid foods* **3**—*only solid foods or just nutritional supplements* **4**—*very little amount of any food* and **5**—*only tube or vein feeding”*. The frequency of each category of food Intake in the previous month vs. usual intake was **0**−5%, 1–2%, 2–59%, 3–25%, 4–6%, and 5–3%.

The reported alterations in food intake were associated with various patient-reported symptoms during the past two weeks: 98% of patients reported having had at least 1 symptom that prevented them from eating adequately and thus having a nutritional impact.

Thirty seven percent (208/561) of the patients classified their functional activity as impaired (“*I don't feel able to perform most of my activities and stay in bed or sitting less than half the day*”).

At hospital admission, 499/561 (89%) of the patients were classified as moderately or severely malnourished, and the remaining were classified as well nourished.

**Figure 3:**
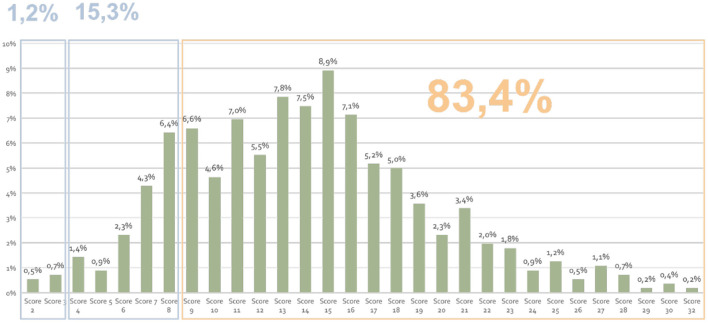
The total numerical score of the PG-SGA.

Eighty three percent (466/561) of the patients scored ≥9 points revealing a critical need for nutritional support. We found that 15% of the patients scored >4 points, indicating the need for directed therapy for symptom control, while only 1% had <2 points (nutritional counseling with pharmacological intervention) (Figure 3).

When focusing only on the three most frequent types of cancers, according to the PG-SGA Global Assessment categories (A, B or C), the prevalence of higher stages (B and C) was observed in patients with colorectal cancer−21% of patients had moderate malnutrition and 2% had severe. Breast cancer had the second highest rate−16% with moderate malnutrition and 2% with severe malnutrition; 8% of gastroesophageal cancer patients had moderate malnutrition and 5% had severe.

Among the patients with a score >9, which addresses the patients in terms of the necessity of urgent nutritional intervention, in these three groups was in concordance with the malnutrition prevalence as previously described: 23% had colorectal cancer, 17% breast cancer and 11% gastroesophageal cancer.

Only 10% of the patients in this cohort had signs of metabolic stress and 7% of them had a high-level of metabolic stress. This corresponds to the metabolic demand described in PG-SGA Worksheet 3. The score for metabolic stress is determined by multiple variables known to increase protein and caloric needs. Note: Score fever intensity or duration, whichever is greater. The score is additive so that a patient who has a fever of 38.8°C (3 points) for <72 h (1 point) and who is on 10 mg of prednisone chronically (2 points) would have an additive score for this section of 5 points.

Almost 54% of the patients had a clinical condition score >2, such as oncologic disease, cardiac or pulmonary cachexia, renal insufficiency, age >65 years and Acquired Immune Deficiency Syndrome (AIDS).

## Discussion

The aim of this study was to characterize the nutritional status of inpatients with cancer at hospital admission, using the scored PG-SGA, in a large series of oncology patients. Typically, patients requiring hospitalization are those with more advanced stages (III/IV), most of them in need of symptomatic control or management of worsening performance status due to greater dependence (29–32).

The scored PG-SGA has shown to be accurate in distinguishing well-nourished patients from malnourished ones. The prevalence of malnutrition in this study was high, with 89% of patients being moderately or severely malnourished. These findings are expected, as patients with cancer have the highest incidence of malnutrition amongst admitted patients (1, 16).

However, at a national level, most centers do not apply nutritional assessment as a routine practice, and the question of malnutrition is oftentimes neglected.

We speculate that there could be barriers to the implementation of the scale, but some data suggest that the patients consider the PG-SGA to be an easy tool to comprehend and the professional component of the PG-SGA received adequate ratings for its content validity, comprehensibility and difficulty (33). Still, the physical exam of the professional component of the PG-SGA usually is the most difficult to understand and use by professionals (33). Studies have shown that significant improvement in PG-SGA-naïve dietitians' perception of comprehensibility and difficulty of the PG-SGA can be achieved quickly by providing 1 day of training in the use of the PG-SGA (34).

This study's results are consistent with findings from similar translations and cultural adaptations of the PG-SGA for Norwegian, Dutch, German and Japanese languages (33–38).

Based on this study's results, and given the large sample size, the patient component of the PG-SGA is ready to be implemented in clinical practice and results confirm that no additional training is needed for patients and professionals to complete their component.

One of the benefits of the PG-SGA is having a part that scores symptoms which may adversely affect nutritional status. In this study, 98% of the patients reported at least one nutrition impact symptom (NIS) that prevented them from eating adequately. Indeed, the prevalence of NIS in this study, was consistent with other studies in patients with cancer (1, 39, 40): the large majority (83%) presented with a score ≥9, indicating the need for urgent nutritional intervention and symptom control. On the other hand, the recommendations for scores <9 include patient and family education, symptom management, and provision of additional food and/or oral nutrition supplements. By offering early nutritional care, we speculate it may be possible to prevent or delay deterioration in the patient's nutritional status. Thus, timely identification of NIS, e.g., decreased appetite, pain, nausea, vomiting, constipation or diarrhea is essential for early symptom management, contributing to improved diet intake.

Of critical importance is the fact that PG-SGA score correlated with percentage weight loss in the previous six months. This result goes in line with other studies where weight loss has been demonstrated to be a major prognostic indicator of poor survival in cancer patients (11). This fact is not unexpected, since weight loss in the last 1 or 6 months is a part of the PG-SGA. Although, exporting this concept to current practice, until 10% of patients with a recent diagnosis of the oncological disease can present this symptom as first sight and up to 30% to 80% during treatment and disease progression, depending on location and etiology (41). This translates to the evident need for an appropriate assessment, even at an early stage.

Contrasting with these results, using WHO criteria (42), only 13% of patients were classified as being underweight, reflecting the limitations of using only BMI to establish the cut-off points for the risk of undernutrition. This further stresses that normal and overweight cancer patients can be at risk for nutritition.

One of the other findings of this study was that the highest prevalence of nutritional risk in our inpatient population was identified in patients with colorectal cancer (25%), followed by breast cancer (21%) and other gastro-intestinal cancers (14%). However, analyzing the percentage variation over the last six months we can conclude that from these three groups the gastrointestinal followed the colorectal cancer are the ones with more prevalence of malnutrition vs. breast cancer.

The high prevalence of malnutrition in breast cancer patients could be a consequence of a bias in this study population. One of the limitations is the statute of a regional hospital and its limited variability of tumor types treated at the Medical Oncology Department. Furthermore, patients with head and neck and lung cancer are managed by a center of reference at another hospital in Lisbon and the Department of Pneumology, respectively, so our numbers do not correspond to the correct prevalence of the disease in our population.

Also, it is noteworthy that patients admitted to the hospital, as previously mentioned, may have more advanced diseases, and thus may not fully represent the spectrum of oncology patients and their nutritional problems. Because the prevalence of undernutrition in cancer patients is associated with the tumor type, location, stage, and treatment, patient differences in these parameters could have affected the proportion of patients at nutritional risk. Although the time elapsed since cancer diagnosis was not considered in the present analysis it could thus bias the ascertained nutritional risk.

These results agree with previous studies that identified these patient groups as of higher risk for nutritional impairment. Evidence shows that patients with upper gastrointestinal, head and neck cancer and advanced colorectal cancer have a worse prognosis when undernourished (4–6).

In future studies, it will be interesting to evaluate the prevalence of nutritional risk in a larger number of oncology inpatients and outpatients, stratified according to the type of tumor, stage, performance status and other comorbidities to determine the incidence of complications, mortality and response to treatments, and to characterize the costs associated with hospital malnutrition in detail.

Notwithstanding its limitations, this study provides valuable information regarding the prevalence and burden of malnutrition in a set of oncology patients representative of routine clinical practice in Portugal.

The notable strength of the present study is that nutritional assessment was performed within 48 h of hospital admission, which allows for early interventions. In this perspective, early screening and referral at hospital admission have the important purpose of reversing/improving clinical nutritional prognosis through individualized intervention (7, 8), with the possibility of reducing the length of hospital stay, the risk for readmission, morbidity, and mortality (11), as well as improving tolerance to treatment and quality of life (9, 10).

In terms of implication to the current practice, services should be designed to guarantee malnutrition risk screening ensues at the first point of contact and at systematic recesses throughout treatment and care to ensure early intervention is provided to those at risk of malnutrition. Health services should recognize opportunities to insert malnutrition identification and prevention strategies into models of care and support key enablers including the education of all health care professionals.

## Conclusion

Malnutrition incidence in cancer inpatients is high. Early screening is of paramount importance to rapidly identify patients in need of critical intervention, in an attempt to provide the best care to cancer patients and delay clinical deterioration. Understanding the magnitude of the problem and in which groups the greatest need exists is a vital step toward the recognition and management of cancer malnutrition.

## Data availability statement

The raw data supporting the conclusions of this article will be made available by the authors, without undue reservation.

## Ethics statement

The studies involving human participants were reviewed and approved by Comissão de ética para a Saúde Centro Hospitalar Barreiro-Montijo. Written informed consent for participation was not required for this study in accordance with the national legislation and the institutional requirements.

## Author contributions

CT, JL, PR, and DS contributed to conception and design of the study. CT, JL, RG, and IF organized the database. CT and JG performed the statistical analysis. CT wrote the first draft of the manuscript. FO and AM wrote sections of the manuscript. All authors contributed to the article and approved the submitted version.

## Conflict of interest

The authors declare that the research was conducted in the absence of any commercial or financial relationships that could be construed as a potential conflict of interest.

## Publisher's note

All claims expressed in this article are solely those of the authors and do not necessarily represent those of their affiliated organizations, or those of the publisher, the editors and the reviewers. Any product that may be evaluated in this article, or claim that may be made by its manufacturer, is not guaranteed or endorsed by the publisher.
